# Supplementary material for: Are carbon emissions trading and green financial instruments synergistic? -Comprehensive quantitative research based on content analysis
Source: PLoS One. 2024 Mar 7;19(3):e0298601. doi: 10.1371/journal.pone.0298601 (PMC10919601; doi:10.1371/journal.pone.0298601)
Supplement: S1 Data — (ZIP) [file pone.0298601.s001.zip › Data/地级市Z_F工作报告.zip!/╘╞─╧/▒ú╔╜╩╨/└┤╫╘╣1⁄2╓┌║┼í╛┬φ┐╦╩2╛▌═°í┐_2014.docx]

各位代表：

现在，我代表市人民政府向大会报告工作，请予审议，请市政协各位委员提意见。

一、2013：经济社会发展稳中向好

2013年是实施“十二五”规划承前启后的关键之年。市人民政府以科学发展观为指导，认真落实党中央、国务院、省委省政府和市委的决策部署，坚持稳中求进、稳中求好，推动经济社会发展取得明显成效，较好地完成了市人大三届二次会议确定的目标任务。经预测，全市生产总值453亿元，增长14%；规模以上固定资产投资增长30%，社会消费品零售总额增长16%，进出口总额增长23.4%，财政总收入增长19.8%，公共财政预算收入增长20.5%，城镇居民人均可支配收入增长15%，农民人均纯收入增长17%，城镇登记失业率和人口自然增长率分别控制在4.5%和6.1‰以内，居民消费价格总水平上涨3%，单位生产总值能耗下降1.9%。

（一）产业发展成效显著

特色农业稳步发展。预计农业总产值达200亿元、增长10.5%，增加值125亿元、增长8%。粮食生产实现“十连增”，总产137.1万吨、增长2.6%。收购烤烟143.4 万担、香料烟23.63万担，实现产值19.09亿元和2.42亿元，分别增长5.9%和9.7%。向上争取各类农业项目资金5.8亿元、林业项目资金2.24亿元、畜牧项目资金1.08亿元投入农林牧业建设，完成中低产田地改造30.5万亩，继续实施“绿化荒山”行动，完成造林45.2万亩，整治非法加工木炭、木材经营加工、活立木移植三项行动初获成效。甘蔗、蔬菜、核桃、茶叶、咖啡、蚕桑等产业稳步发展。统筹国家、省、市资金8873万元扶持农业龙头企业发展，新增省级龙头企业9户、市级龙头企业28户。农民专业合作社新增160个总计达1268个，荣获“全国示范社”称号3个。新增“云南省名牌农产品”3个，保山小粒咖啡、腾冲红花油茶、龙陵紫皮石斛、昌宁核桃获得国家地理标志证明商标和地理标志保护产品认定，建成全国农业标准化示范区4个，隆阳区潞江镇新寨村、腾冲县马站乡兴华社区被评为“全国第二批一村一品示范村镇”。农村集体经济发展壮大，消除“空壳村”129个。畜牧产业健康发展，建成26个国家级、省级和市级标准化养殖示范场，预计实现肉类总产47万吨、增长11.2%，畜牧业产值80亿元、增长13.2%。

“三大战役”稳步推进。预计第二产业增加值160亿元、增长20%。其中，工业总产值和增加值分别达410亿元和117.4亿元，分别增长25%和20%；建筑业增加值42.6亿元、增长20%。工业项目投资力度加大，非电力工业预计完成投资58亿元、增长28%。规模企业培育成效明显，新增34户达139户。园区经济发展初显强势。基础设施建设得到加强，聚集发展效应初步显现，水长工业园区被列为全省销售收入超百亿元的园区进行培育，龙陵工业园区和昌宁生物资源加工园区新批为省级工业园区。5个省级工业园区预计完成工业增加值56亿元、增长67.8%，基础设施投资达9亿元、增长52%，入园企业固定资产投资达50.5亿元、增长37.2%，建成标准厂房45万平方米、增长38.7%，保山工贸园区轻纺产业园、水长工业园区石材产业园等专业园区项目建设扎实推进。民营经济发展活力增强。民营企业和个体工商达73400户、增长8.9%，从业人员21万、增长8%，上缴税金17亿元、增长20%，可完成增加值198亿元、增长24%，占生产总值43%以上。县域经济发展竞相加速。在全省县域经济考核中，腾冲跻身2012年科学发展10强县并位列第6名，隆阳和龙陵位次有较大提升，五县区共同发展的态势初步形成。2013年1—3季度，隆阳经济总量在全省129个县区和24个市（区）中均居第13位；腾冲在25个边境县中经济总量继续保持第2位，增速居第9位。预计隆阳、施甸、腾冲、龙陵、昌宁生产总值分别增长14%、15%、16%、16%和13%，各项主要经济指标均保持两位数增长。

第三产业稳步增长。预计实现增加值168亿元、增长14%。三张旅游名片建设规划编制工作进展顺利，腾冲旅游标准化全国试点县建设通过验收，火山热海5A级景区创建通过省级专家初评，跨境旅游异地办证获得国家批准。旅游重大项目建设稳步推进，腾冲玛御谷悦椿温泉村、云峰山养生度假村、龙陵珠宝城一期等一批项目建成运营，蒲缥温泉旅游度假区等项目建设已近尾声。全年预计旅游总人数950万人次、增长18%，旅游总收入62亿元、增长21.2%。加强现代市场体系建设，农村集贸市场、“南菜北运”试点和“万村千乡市场”等工程建设进展顺利，“农家店”覆盖所有乡镇和80%的村，交通运输、房地产、电子商务等产业平稳较快发展，实现社会消费品零售总额139亿元。

财税金融平稳运行。预计完成国税收入24.77亿元、增长17.3%，地税收入34.68亿元、增长18%；财政总收入68.8亿元，其中公共预算收入42.8亿元；公共财政预算支出150.3亿元。融资平台建设得到加强，市电力公司4.8亿元企业债券、市国资公司23亿元公司债券发行报批工作取得实质性进展，市县两级土地收储中心获批进入融资平台，银行各项融资总额达400亿元。

（二）项目投资再创新高

预计完成规模以上固定资产投资290亿元，交通、能源、农田水利等基础设施建设成效突出。全年投入交通基础设施建设23.36亿元，新改建农村公路1111公里，新增农村公路193公里。农网改造升级和无电地区电力建设通过总体验收，槟榔江三岔河水库建设扎实推进，龙川江梯级电站腾龙桥二级项目已经开工，预计完成规模以上能源投资19亿元、增长33.8%。水利建设投资达20.3亿元、增长22%，段家坝等5座中型水库和善洲水库等2座小型水库、中心城市东河治理、西水东调工程建设进展顺利，龙陵三岔河水库通过省级验收，建成1万口“爱心水窖”，新建2.5万件“五小水利”工程，解决了15万人饮水安全问题。保腾铁路、昌保高速等项目前期工作正在抓紧推进。

（三）城乡建设风生水起

分层完善城乡规划体系，中心城市总规修编已获省政府批复，中心城市发展框架基本形成，主干道建设渐行渐近，会展中心等一批重大项目建成投入使用，中心城市创建国家交通管理模范城市通过验收，打破行政区域集中安置被征地群众的统筹城乡发展新模式取得新鲜经验。4个县城建设各有推进，总规修改、功能配套、风貌统筹、重点项目建设齐头并进。10个省级和27个市级特色小镇建设进展顺利，和顺镇被列为全国第一批美丽宜居小镇，水碓村入选全国最美乡村。59个村庄进入中国传统村落名录，为全省最多州市。启动农村垃圾处理项目，整合资金10. 85 亿元，实施各级美丽乡村试点建设669个。投入各级扶贫资金7.32亿元，组织实施整村推进项目368个，启动整乡推进项目5个，近10万农村贫困人口实现脱贫。城乡统筹转户力度不断加大，完成农业人口转户进城9.6万人。全市新增城镇建成区面积2.1平方公里，城镇化率达38.3%，比上年提高3.16个百分点。

（四）改革开放不断深化

深入推进农村综合改革、财税改革和投融资体制改革，有序推进事业单位分类改革和教育、卫生、文化等社会事业及公共服务领域改革。扎实推进园区实体化管理，进一步理顺市区事权关系，完善中心城市规划建设管理体制，健全市对县区和市直单位及公务员管理考核机制。拓展对缅农业、交通、电力、贸易等合作，在腾冲举办首届边境贸易交易会，对外贸易实现恢复性增长，预计进出口总额1.8亿美元。完成43个桥头堡重大项目投资110亿元，率先组织开展保山参与孟中印缅经济走廊建设研究，《孟中印缅经济走廊保山国际经贸合作示范区总体规划》编制完成并通过专家评审。招商引资实现重大突破，“一把手”带头招商成效明显，专业队伍招商、小分队招商、网络招商迈出新步伐，与义乌松得化纤、深圳卡蒙特服饰、河北迎新玻璃集团等企业签订了一批重大项目投资协议，引进市外到位资金315.2亿元、增长70.5%，引进外资1亿美元、增长37.8%。

（五）民生事业成果丰硕

全市民生类支出115.3亿元、增长10.1%，占公共财政预算支出的76.7%，比上年增加2.8个百分点。稳步实施收入倍增计划，城镇居民人均可支配收入、农民人均纯收入分别达21700元和6240元，增幅高于全市经济增长水平。食品药品监管进一步加强，“永子”等9件商标成为云南省著名商标，康丰糖业公司荣获首届云南省政府质量奖，市场物价总体平稳。各级各类教育健康发展，“两基”水平巩固提升，高中阶段教育毛入学率达69%，高考上线率连续7年高于全省平均水平，实现700分以上零的突破。教育“两免一补”和营养改善补助政策全面落实，校安校改工程和教育园区建设有力推进。科技创新工程建设力度加大，创新平台建设取得实效，获得省级重点新产品认定3个。创建国家公共文化服务体系示范区和全国残疾人文化体育示范市通过验收，杨善洲精神教育基地被列为国家、省重点培训教育基地，滇西抗战纪念馆、杨善洲事迹陈列室建成开放，保山历史名人堂布展工作即将完成，松山抗战遗址公园建设取得阶段性成果，永子棋院开工建设。文化遗产保护与开发利用力度加大，新增3个国家级文保单位和4个省级非物质文化遗产项目。实现文化产业增加值30亿元，占生产总值的6.6%。重大疾病防控和医疗卫生服务体系建设得到加强，基本公共卫生服务均等化水平稳步提高，全民基本医疗保障制度初步建立，新农合参合率达97.4 %。全民健身运动和竞技体育协调发展，成功举办全国第二届史迪威公路汽车越野赛、省第二届宗教界运动会、全市首届残疾人运动会和围棋赛等赛事。就业创业工作全面加强，开发就业岗位2.2万个，城镇新增就业1.58万人，转移农村劳动力51万人次。社会保障体系不断完善，城镇基本养老、医疗、失业、工伤、生育保险参保人数持续增加，参保率均达95%以上。社会救助体系进一步完善，困难群体基本生活得到有效保障。住房公积金制度覆盖9万人，缴存总额突破40亿元。兑现涉农补贴资金3.58亿元，完成国家农户科学储粮工程3万户。开工建设保障性住房2万套，基本建成9000套，实施农村危房改造2.63万户。认真贯彻党的民族宗教政策，全面促进民族团结稳定，不断加快少数民族地区发展。防灾减灾体系日趋完善，受灾群众得到妥善安置。国防建设不断加强，军民关系更加融洽。人口计生、妇女儿童、地方史志、侨务、档案、人防、气象、水文、地震、老龄等工作健康发展。

（六）社会管理再现亮点

切实加强精神文明和民主法制建设，不断强化基层基础工作，完善社会治安防控体系，落实维护社会稳定责任，依法打击各种违法犯罪，“平安保山”、“法治保山”建设扎实推进，“城市一张网、农村一道岗、边境一面墙”创建工程和构建大调解工作格局的“施甸经验”、昌宁“十户联防”模式成为全省推广的先进经验，毒品、艾滋病蔓延势头得到有效遏制，社会公众安全感满意率位居全省前列。深入实施群众工作“六项制度”，综合运用信访、调解、心理疏导、行政复议等多种方式化解社会矛盾，群众信访总量、集体访连续三年下降。不断完善公共突发事件应急预警机制，橄榄河、玉地里等重点地质灾害隐患区专项治理取得显著成效。严格落实安全生产责任，全年未发生重特大安全事故。

（七）政府建设得到加强

认真贯彻中央“八项规定”和省委、市委实施办法，深入开展“四群”教育、向杨善洲同志学习和产业建设年、服务基层年、项目落地年、作风转变年等活动，制度规定得到贯彻落实，各项工作取得明显成效，干部作风有新的改进。切实抓好省委巡视组反馈意见所提问题的整改，积极转变政府职能，加强行政效能建设，完善政府工作规则，学习型、创新型、效能型、服务型、法治型、清廉型政府建设深入推进。严格落实廉政责任，强化行政监察和审计监督，全面加强行政成本控制，会议、文件得到有效精简，公务用车、因公出国（境）管理和公务接待更加规范，全市“三公”经费支出下降了8.8%。督查问责和政风行风热线工作力度进一步加大。依法接受市人大及其常委会的法律监督、工作监督和市政协的民主监督，自觉向市人大及其常委会报告工作，向市政协通报情况，认真听取各民主党派、工商联和无党派人士的意见建议，进一步发挥工会、共青团、妇联等群团组织的桥梁纽带作用，主动接受人民群众、新闻媒体和社会各界监督。全年共办理人大代表建议106件、政协提案203件，办复率、面商率均为100%。政务服务体系建设初见成效，市县（区）政务服务中心、72个乡镇（街道）为民服务中心全面建成并有效运行，电子政务公文交换系统正式启用，政府信息公开工作扎实开展。

各位代表，上述成绩来之不易，这是省委、省政府和市委正确领导、科学决策的结果，是市人大、市政协监督支持的结果，是全市各族人民团结奋斗、开拓进取的结果。在此，我代表市人民政府，向全市各族人民，向人大代表、政协委员，向各民主党派、工商联、无党派人士和各人民团体，向驻保解放军指战员、武警部队官兵和公安政法干警，向离退休老同志，向所有关心支持保山发展的同志们、朋友们致以深深的敬意和衷心的感谢！

在肯定成绩的同时，我们也清醒地看到，我们还面临诸多困难和问题。困难主要表现在：世界经济整体复苏缓慢所导致的不确定性依然存在，国内经济正处于增长速度换挡期、结构调整阵痛期、前期刺激政策消化期三期叠加所带来的经济下行压力依然较大，消费拉动增长依然动力不足，资金、技术、人才等要素制约依然突出，市内交通和国际通道不畅的瓶颈制约依然存在，财政增收和城乡居民增收压力依然很大，我们依然面临扩大经济总量与调结构转方式的双重任务。问题主要表现在：部分干部思想、作风、能力与深化改革、扩大开放、加快发展的要求不相适应，政府工作中高效抓落实、强力抓督查、及时抓问效、科学抓考评等方面尚未建立起完善的机制，政府自身建设仍需加强。以困难为破解对象，以问题为工作导向，克难奋进，破题前行，我们才能不断开创科学发展和谐发展跨越发展的新局面。

二、2014：改革开放发展稳中求快

2014年，是全面深化改革的起步之年，是全力扩大开放的重要之年，也是全面实现“十二五”规划目标的关键之年。今年的主要任务，就是抓改革、扩开放、促稳定、推发展、惠民生，我们要把改革与稳定、开放与发展辩证地统一起来，努力做到稳有定力、改有动力、开放有张力、发展有活力。根据党的十八大、十八届三中全会和中央经济工作会议精神，按照省委、省政府和市委的决策部署，今年政府工作的总要求是：以科学发展观为指导，坚持稳中求进，力争稳中求快，突出跨越发展，全面深化改革，全力扩大开放，抢抓丝路经济带和孟中印缅经济走廊建设的机遇，更加突出转方式调结构，更加突出激发市场活力，更加突出发展外向型经济，更加突出推进特色城镇化，更加突出保障和改善民生，更加突出环境保护和生态文明建设，深入实施“六大战略”，全面落实“四化五加强”举措，聚力打好“三大战役”， 竭力打造三张旅游名片，加快培育文化品牌，切实提高发展质量和综合效益，奋力推动保山经济社会更好更快发展。

2014年经济社会发展主要指标建议为：生产总值增长13%以上，规模以上固定资产投资增长25%以上，社会消费品零售总额增长15%以上，进出口总额增长15%以上，公共财政预算收入增长12%以上，城镇居民人均可支配收入增长13%以上，农民人均纯收入增长16%以上，城镇登记失业率控制在4.5%以内，居民消费价格涨幅控制在3.5%以内，单位生产总值能耗下降1.8%以上。

围绕上述目标，重点抓好十个方面的工作：

（一）努力打造高原特色农业，促进农村经济持续发展

构建农业产业体系。稳步提升粮油、“两烟”、甘蔗、茶叶、咖啡、甜柿、蚕桑等传统产业；大力发展畜牧、核桃、冬早蔬菜、红花油茶、澳洲坚果、草果等高原特色优势产业；加快培育石斛、中药材、食用菌、糯橄榄等新兴产业；聚力打造隆阳区国家级现代农业示范园，积极发展现代农业。抓好粮安工程建设，确保完成总产139万吨。巩固现代烟草农业，发展特色烟叶，提升单产效益，完成收购烤烟134.2万担、香料烟23.75万担，确保收购量减但收入和效益不减。完善畜牧技术服务体系，强化科技推广和疫病防控，推进标准化规模化养殖，实现肉类总产52万吨，畜牧业总产值90亿元。第一产业增加值增长6%以上。

培育新型经营主体。发展壮大家庭农场、农业企业、专业大户、农业庄园、农民专业合作社等新型农业经营主体，力争获批建设省级精品庄园8个，新增农民专业合作社220个。积极探索金融支持农业经营主体的体制机制，建立政府主导的投融资担保平台。培育壮大龙头企业，新增销售收入上亿元企业5户、省级龙头企业6户、市级龙头企业20户。加快发展农村集体经济，消除“空壳村”100个。加强农产品标准化认证和地理标志认证工作，支持龙头企业争创云南著名商标和中国驰名商标，加强优势特色农产品营销，着力打造农林畜产品品牌。

夯实农村发展基础。切实抓好以公路、农田水利、能源、生态环保等为重点的农村基础设施建设，不断提高农业综合生产能力，改善农村生产生活条件。加强重点水源建设，加快推进花园、红谷田、段家坝3座中型水库及西水东调工程建设步伐，开工建设25座小（二）型病险水库除险加固、9条中小河流治理及高效节水灌溉、爱心水窖、水土保持等工程，力争开工建设腾冲小地方、隆阳上坪河等水库。继续实施好中低产田地改造、烟水配套、农业综合开发、土地整理等项目。力争实施120个建制村畅通工程，建成基本农田2.1万亩，高标准农田3.45万亩，改造中低产田地20.4万亩，新增有效灌溉面积9万亩，治理水土流失面积110平方公里，解决15万农村人口饮水安全问题。

抓好扶贫开发工作。坚持以边远山区、少数民族聚居区和深度贫困群体为重点，大力实施整乡开发和整村推进，突出抓好基础设施建设、优势产业培育、社会事业发展等工程，继续加大投入，实施精准扶贫，集中力量打好片区扶贫攻坚战。投入市级以上各类扶贫资金6亿元以上，力争贫困人口减少8万人。

（二）努力扩大工业规模，实现工业经济发展新突破

壮大园区经济实力。以园区为平台，以项目为抓手，深入落实倍增计划，确保全市工业总产值和工业增加值分别增长22%和20%以上。完善五大工业园区产业发展规划，深入落实实体化管理体制，全力打造工业经济发展的“大平台”和“主战场”。着力加强园区承载能力建设，突破配套设施瓶颈，新建标准厂房40万平方米。切实抓好工贸园区轻纺产业园和木材加工交易园、水长园区石材产业园及腾冲综合物流产业园等专业园区建设，努力为中小企业入园发展创造条件。加大招商引资力度，新增入园企业28户以上，5个省级工业园区实现工业总产值210亿元以上。

强化工业项目投资。加快龙陵极斛公司石斛基地及加工厂、昌宁摩尔农庄核桃综合开发、缤纷实业石材加工、复烤厂异地搬迁等项目进度，力争年内建成投产。抓好博翔科技年产6000吨弹性纤维、深圳卡蒙特服饰、鸿博矿业石材加工等项目落地并尽快开工建设，加强100万吨聚酯切片、玄武岩纤维、陶瓷卫生洁具等项目前期工作并争取尽早落地。

抓实要素协调保障。充分发挥保山电网优势，加强两网协调融合管理，保障全市电力输送畅通和企业生产用电需求。加快中缅天然气管道支线及城市管网建设和综合开发利用。进一步加强煤、电、油、运等生产要素协调保障，对符合产业政策导向、暂时经营困难的企业，给予一定期限内缓缴社会保险费的扶持。继续抓好规模以上企业培育，确保年内新增30户。

加快民营经济发展。坚持权利平等、机会平等、规则平等，加大民营经济发展扶持力度，加快中小企业公共服务平台建设，建立企业减负长效机制，切实加强信息、管理、融资、人才、培训等服务，确保民营经济增加值占全市生产总值达45%以上。

（三）努力增加消费需求，推动服务业全面发展

力推旅游文化产业。积极推进旅游与文化、城镇、生态、产业融合发展，加快旅游业转型升级和国际化进程。完成三张旅游名片规划编制并付诸实施，力争火山热海国家5A级景区创建成功，启动北海湿地和云峰山4A级、善洲林场3A级景区创建工作。加快推进特色旅游小镇建设，大力发展生态及乡村旅游，扶持一批特色旅游村、旅游农庄，规范提升“农家乐”经营服务的水平。加快旅游信息化建设，规范发展网上营销，鼓励发展智慧旅游。深化腾冲旅游综合试点改革，推进腾冲至缅甸密支那无障碍跨境旅游合作区建设。深入贯彻实施《旅游法》，加强行业管理，规范市场秩序，提升服务质量，确保旅游总人数、总收入分别增长10%和20%。编制实施五大文化品牌建设规划，抓紧完善文化产业政策，推进国有经营性文化单位转企改制，扶持壮大一批文化企业，确保文化产业增加值占生产总值比重达7%以上。

积极扩大消费需求。认真落实国家促进消费和收入分配调整的政策措施，不断提高城乡居民消费预期，增强社会消费能力和意愿。努力促进餐饮、住宿、购物等服务性消费增长，着力培育住房、教育、文化、医疗等消费热点，大力发展社区商业、物业、家政、养老等便民服务，加快形成一批拉动力强的消费增长点，确保社会消费品零售总额增长15%以上。

发展现代服务经济。审定中心城市商贸物流规划并付诸实施，优化综合超市、农贸市场、批发市场等商业网点布局，推进保山工贸园区现代物流产业园、腾冲中和物流园等项目建设，加快培育金融保险、信息服务、物流快递、电子商务等产业，着力打造滇西现代物流中心，确保第三产业增加值增长14%以上。

（四）努力推进特色城镇化，推动城乡统筹发展

编制完善规划体系。坚持以规划为龙头，严守耕地保护红线，一张图纸调控，切实提高用地集约化程度。扎实开展中心城市空间风貌规划、坝区城乡一体化规划和低丘缓坡土地综合开发利用试点等规划编制工作，推动城镇上山组团发展，形成生产空间集约高效、生活空间宜居适度、生态空间山清水秀的格局。

建设特色宜居城镇。中心城市围绕打造“生态园林、休闲度假、商贸物流”为一体的滇西城市群核心城市和“最适宜人居城市”目标，深入落实规划建设管理体制机制，确保义乌国际商贸城一期建成营运，加快推进五洲国际广场等城市综合体建设。加快“三校一院”、永子棋院、“一湖四路”、沙丙路等重点项目进度，开工建设东环路、北城路等新区道路，切实抓好永昌路、保岫路、青堡路及其综合管沟建设，实施人民路改造工程，抓好红庙等安置小区建设，大力发展公共交通，不断完善城市功能，提升综合承载能力。腾冲以建设国际化旅游城市为目标，启动实施中和新城区建设，完善西山坝新城区、高黎贡山国际旅游城规划建设和综合配套设施建设，加快撤县设市步伐。推进龙陵“山水玉”特色旅游城市、施甸和昌宁山水田园城市建设。支持腾冲特色小镇建设，抓好姚关、勐糯、柯街卡斯等城乡统筹发展试点工作，增强示范带动能力。坚持以人为本，以进城农民市民化为核心，推进农民工融入城镇，切实做好8万以上农业人口向城镇转移，确保城乡一体化发展取得新突破，城镇化率达40.5%。

加强城市综合管理。积极推广中心城市打破行政区域集中安置的经验，加大土地收储力度，盘活城市土地资源，增强融资能力。进一步加强综合监管，强化日常维护，加大执法力度，实现高效管理。大力实施绿化、净化、美化、亮化工程，深入开展违法建设、占道经营、乱停乱放、尘土飞扬、噪音污染等专项整治行动，努力营造整洁优美、畅通有序、文明和谐的城市环境。

推进美丽乡村建设。以“中心村”、“特色村”为重点，注重挖掘民俗文化，保留历史风貌，改善人居环境，提升建设水平，彰显地方特色，打造新农村升级版。有序引导散户并村、迁村并点，积极推动条件成熟的乡改镇、镇改街道、村改社区。加强美丽宜居小镇、美丽宜居村庄、中国传统村落规划建设和申报，建设各类美丽乡村试点500个，重点打造40个市级示范点。

（五）努力强化项目支撑，不断增强发展后劲

加大项目建设力度。严格落实投资工作责任制、重大项目挂钩制，强化项目督查，突出解决“三难”问题，强力推进交通、能源、工业、农业农村、服务业、市政等领域的基础设施建设，确保规模以上固定资产投资增长25%以上。重点抓好现代综合交通运输体系建设，加快大瑞铁路保山段、龙江特大桥、龙瑞高速公路龙陵段、沙（坝）蒲（缥）公路等项目进度，推进通乡油路和农村公路通畅工程。加强能源建设，重点抓好腾冲槟榔江三岔河龙头水库及电站、龙川江龙头水库及梯级电站、农网改造升级、跨境电力贸易等项目，进一步抓好移民安置工作。

抓实项目前期工作。加大重点项目前期投入，提高项目成熟度，充实项目储备库，推进投资项目集中审批、并联审批平台建设，提高审批效率，力争一批重大项目获得国家和省核准并开工建设。重点抓好大瑞铁路保山至瑞丽段、保腾铁路、中猴高速、220千伏兰城变电站和500千伏永昌变电站等项目前期工作。

积极拓宽融资渠道。深入研究国家产业政策，准确把握投资方向，加强对上沟通衔接，力争得到国家和省更多支持。加大直接融资力度，积极支持有条件的企业上市及“新三板”市场融资，做大做强永昌投资公司，落实市国资公司23亿元公司债券、市电力公司4.8亿元企业债券发行和市土地收储中心5亿元融资。进一步扩大企业投资自主权，规范政府投资行为，鼓励支持民间投资，实现投资渠道多元化。

（六）努力推进改革开放，激发更大发展活力

推进重点领域改革。坚持以改革统领全局，以改革促创新发展，以改革促结构优化，以改革促民生改善，力争在新一轮改革开放中赢得主动。认真贯彻落实市委三届四次全会精神，以坚定的信心、坚决的态度、坚韧的干劲、坚实的举措，扎实抓好沿边金融开放、政府职能和机构、投融资体制、社会治理模式等重点领域和关键环节的改革。进一步加强国有资产监管，深化国有企业改革，完善现代企业制度。健全现代市场体系，稳妥推进以电力、天然气等为主的资源性产品价格和管理体制改革。加强农业农村综合改革和制度创新，扎实做好农村建设用地确权登记发证工作，积极推进农村土地承包经营权抵押担保。

拓展对外开放空间。抓住建设丝路经济带和孟中印缅经济走廊的机遇，尽快制定和完善规划，积极争取设立国际经济合作区，力争猴桥边境经济合作区升格为国家级边境经济合作区。坚持亲、诚、惠、容理念，加强对缅关系协调，努力促进政策沟通、道路联通、油气贯通、电讯互通、电力输通、商贸畅通、货币流通、民心相通，加快推进境外陆水联运通道建设，全力打通孟中印缅经济走廊北线通道，做好腾冲口岸机场申报争取工作，为建设国际经济走廊打好基础。鼓励支持企业到境外合作开发交通、农业、水电、矿产及教育、卫生等，推动沿边经济合作区建设。

提高招商引资水平。继续加强“一把手”带头招商，推进县区、园区和部门联动招商，以硬手段打造软环境，为项目引进和落地提供优质服务，努力形成招商引资强大合力，着力引进世界500强和国内500强等大企业，着力引进战略性新兴产业，着力引进外向型企业，确保全年引进市外到位资金增长30%以上。

实现进出口平稳增长。积极争取保税仓库、保税物流中心等口岸基础设施建设，提高通关便利化水平。加快黑泥塘边民互市区建设，争取免税店建成运营。加大出口企业扶持力度，整治违规收费行为，研究出台新一轮出口奖励政策。积极向上争取境外替代种植农产品进口返销指标和项目补助资金，加强对重点出口企业、重点出口业务的跟踪服务，进出口总额增长15%以上。

（七）努力抓好财税金融工作，增强支持保障能力

高度重视财源建设。充分发挥财税政策和财政资金的杠杆作用，重点扶持工业园区、重点产业、重点项目、重点企业，积极培植新兴财源，增强财政增收后劲。坚持依法治税，推进税制改革，强化税收征管，确保公共财政预算收入增长12%以上。

切实强化财政管理。深化预算改革，强化财政管理，硬化预算约束，进一步增强财政宏观调控能力。廓清各级政府支出责任，综合考虑财力与事权的匹配性。统筹各项财政资金，进一步盘活存量，积极稳妥推进预算信息公开。把短期应对措施和长期制度建设结合起来，认真做好防控和化解政府性债务风险的各项工作。建立健全财政绩效评价机制，提高财政资金使用效益。

加大金融支持力度。加强政策引导，增强金融服务实体经济的能力，确保银行各项融资增加67亿元以上，直接融资12亿元以上。用好用足云南建设沿边金融综合改革试验区各项政策，推进沿边金融、跨境金融、地方金融改革创新，力争在人民币跨境结算、金融招商等方面取得突破。创新农村金融产品和服务方式，建立农村产权确权登记、评估、流转交易服务平台，扩大农业保险品种和规模，推进农村信用体系和支付体系建设。

（八）努力加强生态文明建设，提升保山的美丽指数

加快“森林保山”建设。加大宣传教育力度，强化全民生态文明意识，加快发展绿色经济、循环经济、低碳经济，积极推进资源节约型、环境友好型社会建设，努力实现生物生态、生活生态和生产生态相结合，经济效益、生态效益和社会效益相统一，提升保山美丽指数和幸福指数。大力实施绿化荒山行动，巩固退耕还林成果，切实抓好水土流失、石漠化区域、“两江四路”生态恢复治理工程，绿化荒山115万亩，改造低效林36万亩，在低热河谷生态脆弱区规划发展20万亩糯橄榄并在年内完成种植6万亩。按园林城市标准开展城市绿化，确保城市建成区绿化率达33%以上。继续开展林业“三项清理整治行动”，严格森林资源管理，抓好森林防火，强化林政执法，依法打击涉林违法行为。

加强生态环境保护。坚持依法保护生态环境，严格实行生态环境损害赔偿制度和责任终身追究制度，建立完善资源有偿使用和生态补偿机制，促进昌宁城市田园风光、龙陵黄龙玉资源省级立法保护。积极推进生态市创建，强化森林、草原、湿地、野生动植物等资源和生物多样性保护，抓好高黎贡山国家公园建设，加大饮用水源地、湖库、河流、大气、噪声等监测管理和综合整治力度，切实减少环境污染。稳步推进殡葬制度改革，加快农村公墓和骨灰堂等设施建设，极力消除乱埋乱葬现象。

强化节能减排工作。围绕省控目标，强化监控管理，全面完成节能减排任务。大力推广城市清洁能源，推动生活垃圾分类收集、无害化处理设施建设，加快资源化利用步伐，确保城市污水处理率和生活垃圾处理率分别达73%和98.7%以上。

（九）努力保障和改善民生，推动各项社会事业发展

千方百计扩大就业。坚持实施积极的就业政策，多渠道开发就业岗位，鼓励自主创业和自谋职业，重点做好高校毕业生、农民工、下岗失业人员、城镇就业困难人员的就业培训指导工作。开发就业岗位2.2万个，新增城镇就业1.6万人，转移农村劳动力50万人次，城镇登记失业率控制在4.5%以内。

完善社会保障体系。进一步完善城镇基本养老、医疗、失业、工伤、生育保险制度，扩大城乡居民养老保险覆盖范围。切实解决好困难群体基本生活问题，确保城乡低保、农村五保和孤儿保障标准及补助水平提高15%。加强保障性住房建设和管理，加快推进棚户区改造，新开工建设1万套保障性住房。扩大住房公积金制度覆盖面，逐步放宽提取条件，让更多职工平等享有保障权益。健全完善社会救助制度，规范城乡低保动态管理。大力培育养老服务业，健全社会福利和残疾人社会保障服务体系。

促进城乡居民增收。抓好强农惠农富农政策兑现，落实深化收入分配制度改革政策，继续实施居民收入倍增计划，确保城镇居民人均可支配收入和农民人均纯收入分别增长13%和16%以上。

切实加强市场监管。深入推进“品质保山”建设，着力创建“全国质量强市示范城市”。更加重视农产品质量和食品安全，完善统一的食品药品安全监管体系，加强源头治理和产销全程监管，确保人民群众“舌尖上的安全”。综合运用最低收购价、临时收储、政策性保险等手段，有效保障物资供应，满足消费市场需求。努力改进市场监管，切实加强消费维权，营造放心消费环境，确保居民消费总水平涨幅控制在3.5%以内。

办好人民满意教育。全面巩固“普九”成果，不断提高“两基”水平，推动义务教育均衡发展。扎实抓好高中教育“扩容提质”，加快发展职业教育，确保高中阶段毛入学率达73%。继续支持保山学院和中医药高等专科学校改革发展，不断提高高等教育质量。大力发展农村学前教育，确保学前三年毛入园率达55%。抓好校安校改、中西部校舍改造等工程，教育园区建设完成投资3.5亿元以上。全面落实“两免一补”和营养改善补助政策，解决好进城农民子女接受义务教育问题。积极扶持民办教育、成人教育、老年教育，办好各级老年大学，促进各类教育协调发展。

提高科技创新能力。加快推进创新型保山建设，实施重点科技专项、科技创新平台建设、科技创新创业人才培养引进等工程，建立产学研协同创新机制，加强知识产权保护，提升科技创新能力。加大各级各类人才培养力度，为保山跨越发展提供人才保证。

推动文化繁荣发展。巩固提升国家公共文化服务体系示范区和残疾人文化体育示范市创建成果，建立健全公共文化管理体制、服务机制和保障制度。继续实施好广播电视“村村通”、“户户通”等文化惠民工程，加强文化市场监管，抓好文化遗产保护，提高文化下基层服务水平，力争在文化项目建设、文艺作品创作、对外文化交流合作等方面取得新进展。

提升群众健康水平。继续完善新农合制度，确保参合率达96%以上，稳步提高基本公共卫生服务均等化水平。着力加强医疗卫生服务体系建设，扎实抓好市人民医院、市妇幼保健院、腾冲中医院、龙陵中医院迁建及腾冲县人民医院改扩建工程。加快推进公立医院改革，鼓励引导民营医疗机构健康发展。切实加强中医药服务体系建设，积极争创全国基层中医药工作先进市。落实“单独二胎”政策，促进人口长期均衡发展。加强“七彩云南全民健身”工程建设，大力开展全民健身运动，强化竞技体育人才培养，抓好省运会备战参赛工作，筹办好2014年棋赛、车赛。

继续抓好地方史志、侨务、档案、人防、气象、水文、地震、社科研究等工作，促进各项事业全面发展。

（十）努力创新社会治理模式，不断促进和谐稳定

加强民主法制建设。认真执行市人大及其常委会的决议、决定，依法向市人大及其常委会报告工作，自觉接受监督，积极支持市政协发挥民主协商作用，认真办理人大代表建议和政协提案。支持各民主党派、工商联、无党派人士和工会、共青团、妇联等人民团体开展工作。建立健全居民、村民监督机制，提高城乡社区建设管理水平。深入实施“六五”普法规划，扎实推进“四五”依法治市进程，切实维护社会公平正义。

着力建设精神家园。切实加强社会主义核心价值体系建设，推动社会主义精神文明建设与开展感恩教育、学习实践中国梦、云南精神、善洲精神相融合，在全社会形成共同的理想信念、强大的精神支柱和基本的道德规范，弘扬时代主旋律，增加社会正能量，塑造社会正形象，培养文明新风尚。

创新社会治理方式。坚持系统治理、依法治理、综合治理和源头治理相结合的原则，努力实现政府治理和社会自我调节、居民自治的良性互动。加快培育和优先发展经济类、慈善类、公益类、服务类社会组织，激发社会组织活力。以网格化管理、社会化服务为方向，积极预防和有效化解社会矛盾，建立矛盾纠纷调处联动机制，健全基层综合服务管理平台，完善重点人群排查、管控、服务、救助工作体系，使群众问题能反映、矛盾能化解、权益有保障。切实做好新形势下信访工作，推行视频接访和联合接访，建立涉法涉诉信访依法终结制度，应用法律手段解决缠访闹访问题。加强社会治安综合治理，依法打击各类违法犯罪，深入开展禁毒防艾人民战争。全面落实党的民族宗教政策，巩固民族团结、宗教和谐的大好局面。积极支持国防和军队建设，提高军民融合发展水平。强化边境管控，维护边境安宁。始终把人民生命安全放在首位，全面落实安全生产“一岗双责”，加大源头排查治理力度，坚决遏制重特大事故发生。加强防灾减灾体系和能力建设，努力保障人民群众生命财产安全。

三、政府建设：转作风树新风清廉高效

“打铁还靠本身硬”，自胜者强。面对今年繁重而艰巨的任务，我们必须进一步加强政府自身建设，以“职能科学、结构优化、廉洁高效、人民满意”为目标，转变作风，狠抓落实，心清身廉，务求高效，全面正确履行职能，塑造政府良好形象。

（一）切实加强理论武装。政府建设，首要的是思想建设。我们必须以习近平总书记系列重要讲话精神武装头脑，进一步坚定理想信念，进一步坚定政治立场，进一步坚守政治纪律，紧密结合保山发展实际和政府工作实际，组织开展好学习、培训和研讨，不断提高各级干部的思想理论水平，进一步增强机遇意识、责任意识、进取意识，不断提升争取支持、推动落实、协调各方、服务发展的能力，为保山改革开放发展奠定坚实的基础。

（二）切实开展群众路线教育。以为民务实清廉为主题，以杨善洲精神为镜子，扎实开展党的群众路线教育实践活动。坚持遵循中央“八项规定”和省委、市委实施办法，以倡勤治懒、倡廉治奢为重点，坚决反对“四风”，大兴联系群众之风、调查研究之风、艰苦奋斗之风、勤俭节约之风。牢固树立“为官避事平生耻”的意识，敢于担当、敢于涉险滩、敢于啃硬骨头，以壮士断腕的勇气、“钉钉子”的精神、“踏石留印、抓铁有痕”的劲头和务实高效的作风狠抓落实。健全政务服务体系，减化办事流程，加强督查问效，努力使行政效率更高、服务环境更优、工作效果更好。

（三）切实转变政府职能。政府职能转变和机构改革既是全面深化改革的“马前卒”，又是宏观调控的“当头炮”。围绕理顺政府与市场、政府与社会的关系，在职能转变上重点抓好“接、放、管”，既要把中央和省下放给市场的权利接转放开，把中央和省下放给地方的职能接好管好，又要把市级政府该放的权利切实放下去，还要把该管的事情管起来、管到位；在机构改革上着力搞好“控、调、改”，既要严格控制机构编制总量，又要调整优化机构编制结构，还要深化改革推动机构编制释放潜力。同时，稳步推进事业单位分类改革，积极探索政事分开、管办分离的有效实现形式。健全行政运行机制，推广政府购买服务，完善发展成果考核评价体系，提高政府管理效率和水平。

（四）切实规范政府行为。增强法制理念和程序意识，推行政府法律顾问制度，坚持重大决策公众参与、专家论证、风险评估和集体讨论决定制度，健全规范性文件及重大决策合法性审查机制，提高政府决策民主化、科学化水平。深化行政执法体制改革，规范行政执法行为，完善行政许可、行政复议、行政赔偿等制度，加强行政执法监督，确保严格公正文明规范执法。进一步加强审计监督、行政监察监督、内部层级监督，自觉接受新闻舆论监督、社会公众监督，不断提高依法行政水平。

（五）切实加强制度约束。严格执行党风廉政建设责任制，全面推进惩治和预防腐败体系建设。坚持用制度管权、管事、管人，推行政府及部门权力清单制度，依法公开权力运行流程，把权力关进制度的笼子里，构建遵循程序、决策科学、执行坚决、监督有力的权力运行体系。继续推进政务、村务和国有企事业单位办事公开，加强政风行风建设，积极回应社会关切，让群众看得到、听得懂、信得过。坚决贯彻《领导干部廉洁从政若干准则》和《党政机关厉行节约反对浪费条例》等制度，勤俭节约办事，艰苦奋斗创业，力控行政成本，继续精会简文，严控“三公”经费和会议经费，严肃查处各种违纪违法行为，树立政府清廉形象。

各位代表！百舸争流，破浪者当先；千帆竞发，奋勇者领航；万马奔腾，跨越者居上；后发先至，保山人在行。前行的路上不会一马平川，赶超跨越必定充满挑战。但是，山高高不过人，路长长不过脚。一切都在变，一切都会变，保山经济社会发展长期向好的大趋势没有变，保山人民盼发展促发展向往美好幸福生活的基本面不会变。只要全市各族人民团结一心，勇于攀登，敢于拼搏，善于进取，就必将凯歌行进、一往无前。让我们紧密团结在以习近平同志为核心的党中央周围，在省委、省政府和市委的坚强领导下，进一步解放思想，锐意进取，克勤克俭，开拓创新，为实现保山经济社会更好更快发展而努力奋斗！
